# Trends in Nanomaterial-Based Non-Invasive Diabetes Sensing Technologies

**DOI:** 10.3390/diagnostics4020027

**Published:** 2014-04-21

**Authors:** Prashanth Makaram, Dawn Owens, Juan Aceros

**Affiliations:** 1 Alpha Szenszor Inc., Carlisle, MA 01741, USA; E-Mail: p.makaram@alphaszenszor.com; 2 Department of Electrical Engineering, University of North Florida, Jacksonville, FL 32246, USA; E-Mail: n00152181@ospreys.unf.edu

**Keywords:** blood, urine, sweat, saliva, tears, breath, diabetes, glucose, sensing, nano-material, monitoring, non-invasive

## Abstract

Blood glucose monitoring is considered the gold standard for diabetes diagnostics and self-monitoring. However, the underlying process is invasive and highly uncomfortable for patients. Furthermore, the process must be completed several times a day to successfully manage the disease, which greatly contributes to the massive need for non-invasive monitoring options. Human serums, such as saliva, sweat, breath, urine and tears, contain traces of glucose and are easily accessible. Therefore, they allow minimal to non-invasive glucose monitoring, making them attractive alternatives to blood measurements. Numerous developments regarding noninvasive glucose detection techniques have taken place over the years, but recently, they have gained recognition as viable alternatives, due to the advent of nanotechnology-based sensors. Such sensors are optimal for testing the amount of glucose in serums other than blood thanks to their enhanced sensitivity and selectivity ranges, in addition to their size and compatibility with electronic circuitry. These nanotechnology approaches are rapidly evolving, and new techniques are constantly emerging. Hence, this manuscript aims to review current and future nanomaterial-based technologies utilizing saliva, sweat, breath and tears as a diagnostic medium for diabetes monitoring.

## 1. Introduction

Diabetes is one of the most prevalent and pressing diseases in the world today. It is caused by an insulin disorder that can be classified as type 1 or type 2 according to its underlying mechanism. Type 1 (also known as insulin-dependent diabetes or juvenile diabetes) results from an autoimmune attack destroying the insulin producing beta cells of the pancreas. Type 2 (also known as non-insulin dependent diabetes or adult onset diabetes) is a metabolic disorder characterized by high blood glucose involving insulin resistance and relative insulin deficiency. Additionally, high blood sugar levels during pregnancy causes a third type of diabetes, gestation diabetes mellitus (GDM). Women with GDM and their babies are at a high risk of developing type 2 diabetes within 5–10 years after delivery.

According the World Health Organization (WHO), 347 million people worldwide suffer from diabetes [1], with type 2 diabetes accounting for approximately 90% of all cases [2]. Furthermore, there are 183 million [3] people worldwide that are currently undiagnosed and able to benefit from affordable and convenient early detection technology. Such detection technology is beneficial not just for the diagnosis of the disease, but also for its monitoring and management, therefore helping diminish its serious health and life-threatening side effects. Because of such a high impact to the quality of human life, the diabetes diagnostics/therapeutics market is posed to reach $33 B by 2016 [4].

The gold standard for diabetes diagnosing and self-monitoring involves checking the glucose levels in the blood. Such methods consist of using a lancet device that pricks the finger or forearm for a droplet of blood. The droplet is then placed onto a disposable strip containing a sensing element. A glucose meter or glucometer is then used to read blood glucose levels in milligrams per deciliter. Depending on the patient’s condition, hyper- or hypo-glycemic, the glucose levels can be corrected using insulin or glucose, respectively. Even though there have been attempts to reduce the amount of blood required along with miniaturization of the device, the underlying process is still a painful, invasive pricking process, which generally occurs multiple times a day. Hence, there is an immediate need for effective minimal or non-invasive diabetes monitoring.

The following is a brief review of state-of-the-art techniques and the technologies being developed to address this challenge. Some excellent reviews on nanotechnology for glucose detection have been published, but all of them restrict their focus to either nanomedicine [5] or, in the case of Cash *et al*. [6], on nanomaterials specifically aimed at improving current blood-based diagnostics. In this manuscript, we present current and future nanomaterial-based technologies being developed for the minimal to non-invasive monitoring of this disease using breath, saliva, sweat and tears. First, we will review the gold standard for diagnostics and monitoring, followed by our non-invasive sensor review, and finally, we will conclude with future work.

## 2. Blood-Based Diagnostics (The Gold Standard)

Electrochemical transducers are the most widely used blood glucose sensors, due to their sensitivity, reproducibility and manufacturability in huge volume and at low cost [7]. The enzymes most commonly used in the design of glucose biosensors are glucose oxidase (GOx) and glucose dehydrogenase (GDH), which change redox states during the biochemical reaction. The oxidase enzymes accept electrons when they interact with their substrates, and thus change into an inactive state. These enzymes return to their normal active oxidized state by transferring electrons to molecular oxygen, thus producing hydrogen peroxide (H_2_O_2_):

glucose + O_2_ → gluconolactone + H_2_O_2_

Nano-sensors for glucose detection mainly rely on nanomaterials to assist the standard enzymatic electrochemical detection of glucose. Nanomaterials provide advantages, such as a high surface area, enhanced electron transfer from enzyme to electrode and the ability to include additional catalytic steps. For example, nanowires and nanotube arrays made of zinc oxide [8,9,10], ruthenium [11] and gold [12] have been used to increase the surface area with electrochemical reaction improvements compared to conventional electrodes. Similarly, nanoparticles of palladium [13] and gold [14] have been used in membrane configurations to enhance electron transfer. Other nanostructures that have been used to improve sensor performance include conductive polymer blends, which can be operated at varying potentials, thus minimizing the electrochemical interference from common electroactive compounds (e.g., acetaminophen, ascorbic acid and uric acid), which can cause nonspecific signals during sensing. As an example, highly ordered nanotubes of conductive polymers (polyaniline) capable of immobilizing GOx were able to obtain a low detection limit of 0.3 ± 0.1 µM with a range of 0.01–5.5 mM [15].

While sensors based on the above-mentioned materials showed sensitivity in the order of µM–mM, carbon nanotube (CNT)-based sensors have demonstrated enhanced sensitivities in the order of pM–nM with a linear range. The enhanced sensitivity arises from CNT’s unique structure and geometry. The average diameter of single walled CNTs is ~1 nm, and they are composed entirely of surface atoms; thus, molecular adsorbents can significantly alter their electrical properties. This combined with their near-ballistic electron transport properties enables highly sensitive detection. CNTs are usually coupled with other nanomaterials, such as polyaniline, Au and Ag nanoparticles, in order to achieve selectivity. For example, sensors made of CNT/nafion/GOx/Pd nanoparticle paste have demonstrated a detection limit of 0.5 µM glucose with a response time of three seconds [16]. The CNT/nafion/GOx couples the electrocatalytic detection of hydrogen peroxide with the permselectivity of nafion, yielding a highly selective low potential (−0.05 V *vs.* Ag/AgCl) glucose biosensor [17]. CNT-GOx composites were also packed within a 21-gauge needle for amperometric monitoring of glucose. The resulting microsensor yielded highly sensitive and selective devices with a wide linear range (up to 35 mM) and a long lifetime [18]. An extra coating of nafion on Pd-CNT-GOx paste eliminated interferences from common electroactive compounds found in blood [19]. These devices showed linear responses up to 30 mM with a detection limit of 0.6 mM. Lin *et al.* [20] eliminated the need for mediators by covalently immobilizing glucose oxidase CNT via carbodiimide chemistry by forming amide linkages between their amine residues and the carboxylic acid groups present on the CNT. The amperometric sensor operated at a low potential (−0.2 V *vs.* SCE) with linearity up to 30 mM glucose and a detection limit of 0.08 mM. 

While these examples demonstrate the advantages of nano-material sensors in blood based diabetes, it is only a quick snippet of the technology, and the reader is referred to a recent review from Zhu *et al*. [21], which provides a thorough analysis of carbon nanomaterial (carbon nanotubes and graphene)-based sensors for glucose detection. 

## 3. Urine-Based Diagnostics

Urine-based glucose diagnostic tools have been on the market since 1996, predominantly in Asia (Japan) [22]. Urine is one of the most extensively analyzed biological fluids in clinical investigations, due to its high availability and non-invasive collection. In spite of this, samples for biomolecule detection need pre-treatment techniques, because of the low concentration of target analytes, the inherent complexity of the matrices (urine contains non-protein nitrogen metabolites, carbohydrates and proteins, in concentrations of less than 0.6–10 mg/mL (99–166 µM), 44–500µg/mL (1.47–166 µM) and 0.1–20 µg/mL (0.002–0.3 µM), respectively) and even the incompatibility with the instrumentation, which limits the sensitivity and selectivity of the measurement. Several metabolic disorders are characterized by the secretion of abnormally large quantities of sugar(s) in the urine, and diabetes mellitus is one of the most common [23]. 

Ordinarily, glucose and other components are present in urine in trace quantities. Glucose overflows into the urine when the blood glucose level is high, causing urine to have a sweet or fruity odor [24]. The presence of high glucose levels in urine is considered a dangerous condition, as it is an indication of the worsening of diabetes. A positive urine test indicates that the amount of glucose is more than 50–100 mg/dL (2.78–5.55 mM) [25].

A urine glucose meter amperometrically detects glucose by immobilizing a glucose oxidase (GOx) membrane on a peroxide electrode [26]. The system consists of a biosensor and utilizes photolithography technology to fabricate electrodes. Glucose is enzymatically converted to hydrogen peroxide by GOx, yielding hydrogen peroxide, which is then electrochemically detected by the electrodes. 

In 2009, Tanita Corp of Japan introduced the UG-201, which employs a biosensor based on the enzyme electrode method to test glucose levels in urine [27]. The portable urine glucose meter can be carried around with the sensor unit immersed in the preservation solution contained in the cap. The measurement range of the urine sugar level is 0–2000 mg/dL (0–111 µM) and indicated in increments of 10 mg/dL (0.56 µM) at a minimum. The measurement completes in about six seconds and has robustness against the influence of interferents, like ascorbic acid or acetaminophen. However, the system requires weekly calibration in a reference solution for continued use.

Recently, Su *et al*. [25] discovered that ZnFe_2_O_4_ magnetic nanoparticles (MNPs) possess intrinsic peroxidase-like activity, which can be used as a colorimetric biosensor for the detection of urine glucose. ZnFe_2_O_4_ MNPs possess high catalytic efficiency, good stability, monodispersion and rapid separation over other peroxidase nanomimetics and horseradish peroxidase (HRP). ZnFe_2_O_4_ MNPs are inexpensive, highly sensitive and selective for glucose detection using glucose oxidase (GOx) and have a linear range from 1.25 × 10^−6^ to 1.875 × 10^−5^ mol/L (1.25–18 µM) with a detection limit of 3.0 × 10^−7^ mol/L (0.3 µM).

Siyang *et al*. [24] proposed an alternative method to detect diabetes based on direct measurement of urine odor by using an electronic nose (e-nose). To simulate diabetic patients, artificial urine samples were prepared by adding glucose to pure urine samples. Eight commercial chemical gas sensors were used as the sensing elements for the e-nose. Principal components analysis (PCA) and cluster analysis (CA) methods were employed for data analysis. The technique was able to identify the glucose concentration in urine.

Carbon nanotubes have also been studied for the detection of glucose in urine. Zhang *et al.* [28] solubilized (CNT) in aqueous solutions of a biopolymer chitosan (CS), which allowed for interference-free determination of glucose (a detection limit of 3 µM) in urine. When gold-platinum alloy nanoparticle (Au-PTNP) modified CNTs were solubilized in CS films, a lower detection limit of 0.2 µM with a wide range of 0.001–7.0 mM was obtained [29].

## 4. Saliva-Based Diagnostics

While blood and urine are the most commonly used fluids for diagnostic purposes, other biological fluids, such as saliva, sweat and tears, offer some distinctive advantages and are increasingly being considered for diagnostics. In the case of saliva, whole saliva can be collected non-invasively without needing specific equipment or trained personnel. Since the collection of saliva requires less subject compliance, as compared with the collection of blood, its analysis is more valuable for children and older adults. Saliva analysis may also provide a cost-effective approach for the screening of large populations [30].

Saliva can indicate local and systemic alterations, and certain components can be related to the neurologic, metabolic, hormonal and immunologic state of an individual [31]. Saliva tests have already been used for diagnosis purposes, especially for some molecules, such as antibodies, unconjugated steroids, hormones and certain drugs, where the technique has shown sufficient sensitivity to reflect the blood concentration levels of substances accurately [32].

Glucose is a small molecule that is capable of moving easily from blood plasma through the membranes of the blood vessels to the gingival fluid, via the gingival sulcus, reaching the saliva [33]. Thus, an increase in blood glucose in diabetic patients could lead to higher levels of salivary glucose, which, in turn, causes the loss of homeostasis and greater susceptibility to oral cavity diseases [34]. While a number of literature findings suggest a comparative value of blood and saliva glucose levels, there still exists some controversy. In spite of this, Ben-Ayreh *et al.* [35], Aydin [36] and Carda *et al.* [37] have demonstrated that salivary glucose levels in type 2 diabetic patients are much higher than in non-diabetic patients.

Typical saliva glucose levels are in the range of 8 µM to 0.21 mM [38], much lower than blood glucose levels that range from 2 to 30 mM [39]. Thus, most of the tests reported utilize highly sophisticated spectrophotometry to determine glucose concentrations. Recent developments in highly sensitive materials are paving the way for the low-cost easy adoption of these saliva glucose tests. For example, Macaya *et al.* [40] developed electrochemical sensors using a transistor with a channel made out of poly(3,4-ethylenedioxythiophene):poly(styrene sulfonate) (PEDOT:PSS) and a Pt gate electrode with a minimum detection limit of 1 µM. An all-plastic electrochemical sensor was developed using a PEDOT:PSS channel and gate, along with a ferrocene mediator, that showed an adequate response change in the range of 1–200 µM [41].

A challenge in high sensitivity glucose detection is the fact that the device it is a small, weakly charged molecule. Recently, Cella *et al.* [42] used single-walled nanotube (SWNT) chemiresistive sensors to achieve a sensitivity of 1 pM with high selectivity over other sugars and human serum proteins. The detection principal is based on the displacement of plant lectin, concanavalin A, bound to a polysaccharide, dextran, immobilized on SWNTs by the glucose molecules. This sensitivity is higher than that of similar systems that were reported, such as ConA-cyclodextrin/dextran solution-based assays using fluorescence resonance energy transfer between CdTe quantum dots and gold nanoparticles (50 nM) [43] and the infrared absorbance of carbon nanotubes deaggregation (3.7 mM) [44]. Similarly, carbon nanotube FET sensors functionalized with pyrene-1-boronic acid were able to detect glucose in a clinically relevant range of 1 µM–100 mM with a minimum detection limit of 300 nM [45]. These values are significantly higher than the range (0.9–9.1 µM) and detection limit (0.49 µM) of a system constructed of 3-aminophenyl boronic acid and conductive polymer [46]. The high sensitivity can be attributed to the electrostatic transport properties of CNTs. The reaction of boronic acid with glucose molecules results in boronate anions that modulate the electrostatic transport properties of the FET in a concentration-dependent manner [45].

## 5. Sweat-Based Diagnostics

Compared to all non-invasive diagnostic techniques, sweat is the easiest to get access to for sampling, as sweat is readily available in human beings [47]. Recent studies suggest that it is possible to propose a system based on the glucose concentration in sweat to detect blood glucose levels [48]. While normal blood glucose levels range between 90 mg/dL (5 mM) and about 140 mg/dL (7.75 mM) or higher, by contrast, sweat glucose has been reported in significantly lower concentrations of five to 20 mg/dL (0.277–1.11 mM) [49].

Artificially drawing tissue fluid through the skin has been achieved through a process called reverse iontophoresis. Reverse iontophoresis is based on the flow of a low electrical current through the skin, between an anode and cathode positioned on the skin surface. An electric potential is applied between the anode and cathode, thus causing the migration of sodium and chloride ions, which carries water, which, in turn, carries glucose from beneath the skin toward the cathode and anode, respectively. There is a direct correlation between glucose concentration in the physiological fluid and glucose concentration in blood [50]. Even though this method is accurate and a non-invasive technique, there are several limitations, such as a 20-min time lag between fluid extraction and glucose measurement. The accuracy requirement for devices (glucose concentration in this fluid is ~1/1000 that of blood glucose) can be adversely affected, due to prolonged reverse iontophoresis [51].

This technique has been exploited in making the Glucowatch. It uses an electrochemical-enzymatic sensor that is sensitive to the enzyme, glucose oxidase (GOx). The glucose oxidase catalyzes the oxidation of glucose to gluconic acid and hydrogen peroxide. The hydrogen peroxide is detected via an electrocatalytic oxidation reaction at a platinum-containing working electrode in the sensor, where an electric current is produced. For every glucose molecule extracted, two electrons are transferred to the measurement circuit. The magnitude of the resulting electric current is correlated with the amount of glucose collected through the skin [52,53,54]. However, the Glucowatch causes a rash under the skin and is unreliable in detecting hypoglycemia and hyperglycemia [55]. To overcome this issue, Sun *et al.* [56] used a carbon nanotube epoxy composite on a planar screen-printed carbon electrode that demonstrated a fast response (25 s) and good sensitivity (4 μA/mM) with an excellent linear response (*R*^2^ = 0.999; 0–4 mM) at a low detection potential (+500 mV *vs.* Ag/AgCl).

Saraoglu *et al*. [47] used relative variation humidity sensors on 200 subjects and, with the help of an artificial neural network (ANN) architecture, demonstrated a correlation between the glucose concentration value and human palm perspiration. Micro/nano skin systems [57] based on soft-MEMS technologies [58] and nano-structures [59] combined with advanced algorithms can thus be used to measure glucose levels from palm perspiration measurements.

## 6. Tear-Based Diagnostics

Numerous investigations have suggested testing glucose in tear fluid as a substitute for blood, and this concept dates back to the 1950s. If a good correlation between tear glucose and blood glucose samples can be established, the measurement of tear glucose levels could provide an attractive indirect measurement method for blood glucose levels within the normal, as well as hyperglycemic and hypoglycemic ranges [60].

Tears are more accessible than blood or interstitial fluids, more readily available and less susceptible to dilution than urine [61]. Tear fluid is maintained at a miniscule and relatively stable volume (4 µL). Tear fluid is continuously replenished by the production of the lacrimal gland and other accessory glands at a rate of production in the range of 0.5–2.2 µL/min [62].

There has been considerable research focused on the determination of glucose in tears with different methods. For any technique to be analytically useful, it requires a very low detection limit (since glucose is present in tear fluid at levels of 50–100 times lower than in blood), high selectivity over active interferents and the ability to quantitatively measure very small sample volumes in a short time period. Techniques that have been utilized for detection include capillary electrophoresis (CE) coupled with laser-induced fluorescence (LIF) [63], fluorescence sensors [64], liquid chromatography (LC) coupled with electrospray ionization mass spectrometry (ESI-MS) [65], holographic glucose sensors [66], a miniaturized flexible thick-film flow-cell detector [67] and a strip-type flexible biosensor [68].

One of the first reports of a flexible electrochemical biosensor used for glucose monitoring in human tears, saliva and sweat was published in 1995 by Mitsubayashi *et al*. [69], where the bio-device was based on immobilized glucose oxidase. The integration of glucose biosensors into contact lenses has been realized by several research groups. Google Inc. recently announced a contact lens glucose sensor, first developed by Yao *et al.* [70,71] at the University of Washington. Yao integrated functional contact lens, composed of a differential glucose sensor module, metal interconnects, a sensor read-out circuit, an antenna and a telecommunication circuit to monitor the tear glucose levels wirelessly, continuously and non-invasively. The electrochemical differential sensor module is based on the immobilization of activated and de-activated glucose oxidase. Nafion^®^ was used to decrease several potential interferences (ascorbic acid, lactate and urea) present in the tear film. Titania sol-gel film was applied to immobilize glucose oxidase. The sensor had a detection limit of 0.01 mM and showed good linearity or the typical range of glucose concentrations in the tear film (0.1–0.6 mM). Contact lens can be worn for hours without discomfort and, thus, provide an ideal vehicle for non-invasive and continuous glucose monitoring. With different types of contact lenses, functional and practical bionic contact lenses could potentially be realized in the near future. However, one persistent problem for these types of devices is the implementation of a suitable power source.

Researchers at the Fraunhofer Institute for Microelectronic Circuits and Systems (IMS) in Duisburg, Germany, worked with Dutch medical firm Noviosense to produce a low-power biosensor for blood glucose that combines measurement and digital analysis with both an RF link and can be powered by RF energy in the environment. The proclaimed biosensor can continuously measure glucose levels using other tissue fluids, such as sweat or tears. The new biosensor chips is claimed to measure just 0.5 × 2 mm and consume less than 100 µA at 5 V. The device’s chip integrates a nano-potentiostat that measures the concentration of hydrogen peroxide and other chemicals that result from an electrochemical reaction that takes place with the aid of glucose oxidase. The device uses the concentrations of these chemicals to calculate the patient’s glucose level, and through the device’s analog digital data converter and transmitter, the collected data is wirelessly sent to a glucose pump or a compatible smart phone [72]. Qin *et al.* [60,62] employed an amperometric needle-type electrochemical glucose sensor intended for tear glucose measurements in conjunction with a 0.84 mm capillary tube to collect microliter volumes of tear fluid. The sensor was based on immobilizing glucose oxidase on a 0.25 mm platinum/iridium (Pt/Ir) wire, anodically detecting the liberated hydrogen peroxide from the enzymatic reaction. The inner layers of nafion and an electropolymerized fllm of 1,3-diaminobenzene/resorcinol greatly enhanced the selectivity for glucose over potential interferences in tear fluid, including ascorbic acid and uric acid. The sensor achieved a detection limit of 1.5 ± 0.4 μM of glucose with a sensitivity of 0.032 ± 0.02 nA/μM with only 4–5 μL of tear fluid.

Recently, Falk *et al.* [73] developed a microscale membrane-less biofuel cell, capable of generating electrical energy from human lachrymal liquid, by utilizing the ascorbate and oxygen naturally present in tears as the fuel and oxidant. The biodevice is based on three-dimensional nanostructured gold electrodes covered with abiotic (conductive organic complex) and biological (redox enzyme) materials functioning as efficient anodic and cathodic catalysts, respectively. Findings from controlled experimentation support the proposition that an ascorbate/oxygen biofuel cell could be a suitable power source for glucose-sensing contact lenses to be used for continuous health monitoring by diabetes patients. Important challenges that have to be overcome while designing ophthalmic sensors are: (1) the tear lactate concentration is in the range of 1–5 mM, much higher than the concentration in blood (0.5–0.8 mM) [74]; and (2) the pH value in tear fluid is variable (a range of 6.5–7.6) [75]. Yang *et al.* [66] tried to address these challenges by developing polymer-based holographic sensors.

Researchers at Purdue University in Indiana created a non-invasive biosensor that detects minute concentrations of glucose in saliva, tears and urine. The sensor has three main parts: layers of nanosheets resembling tiny rose petals made of graphene, platinum nanoparticles and the enzyme, glucose oxidase. Each petal has a few layers of stacked graphene, with the edges of the petals configured as dangling, incomplete chemical bonds. These defects in the petal edges are by design, since they provide the locations where platinum nanoparticles can attach. Electrodes are formed when the petals on nanosheets combine with the platinum nanoparticles. The glucose oxidase enzyme then attaches to the platinum nanoparticles. When glucose is detected, the enzyme converts the glucose to peroxide, which generates a signal on the electrode [76].

## 7. Breath-Based Diagnostics

Breath tests are gaining popularity as non-invasive testing alternatives for numerous traditional disease diagnoses, due to their quick results and ease of use. Exhaled breath consists of many different molecular species, and their composition gives valuable information about biochemical processes in the body [77,78]. They have proved to be successful in diagnosing diseases [79], like cancer (lung, breast, colorectal) [80,81], *Helicobacter pylori* infection [82,83] and airway inflammations [84,85].

Acetone (2-propanone) is the major ketone in exhaled human breath. It is believed that the fruity odor in the breath increases significantly during periods of glucose deficiency and has long been known as a useful biomarker of type I diabetes. The common understanding is that acetone in human breath is a metabolic product of acetoacetate by the elimination of CO_2_. This reaction is either a result of the non-enzymatic decarboxylation of acetoacetate or is catalyzed by acetoacetate decarboxylase [86].

CH_3_COCH_2_COO^-^ + H^+^ → CH_3_COCH_2_COOH → CH_3_COCH_3_ + CO_2_

This acetone is absorbed into the blood stream and excreted in the breath along with the normal constituents of human exhaled breath. Diabetic patients are either unable to make insulin or the insulin does not work effectively, and the body uses fat instead of glucose for energy, leading to large amounts of acetone in blood and breath. Acetone concentrations in the plasma of diabetic patients were shown to be elevated by at least two orders of magnitude [87,88]. The acetone in expired air from lungs follows the diffusion law and is approximately 1/330 of the acetone in plasma [89].

Most initial studies on detecting breath acetone concentrations have been carried out using expensive and complicated GC-MS, cavity ringdown spectroscopy and selected ion flow tube mass spectroscopy (SIFT-MS). Crofford *et al.* [88] in 1977 carried extensive studies on patients using GC/MS systems and concluded that while acetone is useful as a monitoring tool for the weight reduction program and for diabetic outpatients, it is not useful in following diabetics who are admitted in hospitals with ketoacidosis. An acetone breathe analyzer using cavity ringdown spectroscopy measured acetone concentrations in the range of 0.80 to 3.97 parts per million by volume (ppmv) for four type I diabetic patients (14 h of overnight fasting), higher than the mean acetone concentration (0.49 ppmv) in non-diabetic healthy breath [90]. A follow-up study by the same group showed a direct correlation between BG level (*R* = 0.98, *p* < 0.02) and A1C level (*R* = 0.98, *p* < 0.02) with the breathe acetone concentration for type 1 diabetes when the BG levels grouped as 40–100, 101–150, 151–200 and 201–419 mg/dL and the A1C levels are grouped as <7, 7–9.9 and 10–13 [91]. Selected ion flow tube mass spectrometry (SIFT-MS) was used to monitor the breath of eight patients with type 1 diabetes mellitus during “insulin clamp” studies in which insulin and glucose were infused into patients to lower blood glucose levels. The authors showed that while the initial breath acetone concentration was variable (1 ppm to 21 ppm at 6 mM), the breath acetone level declined linearly with blood glucose concentration [92]. A similar study using SIFT-MS on eight children with type 1 diabetes showed no correlation of 24 volatile organic compounds (VOCs) (including acetone) with blood glucose level [93].

Chakraborathy *et al.* used sonochemically prepared nanosized ϒ-Fe_2_O_3_ sensors that could detect sub-ppm of acetone against a background of simulated human breath [94]. A portable gas analyzing system consisting of an array of conductive polymers (polypyrrole) was developed to discriminate between diabetic patients and normal persons [95]. FETs made out of 10 nm ultrathin InN films has achieved a detection limit of 400 ppb of acetone, demonstrating the potential for diabetes detection in human breath [96]. A chemiresistor based on electrospun metal oxide nanowires [97] and ferroelectric WO_3_ nanoparticles [98,99] have been used to detect acetone selectively in breath-simulated media, paving the way for a diagnostic tool. SnO_2_ thin film sensor arrays were employed by Wang *et al.* [100] to monitor acetone exhaled from the nose of 32 volunteers (18 idiopathic diabetics and 14 non-diabetics), before a meal and at intervals up to 2 h after the meal. They were able to achieve 100% discrimination between diabetic and non-diabetic patients 1 h after the meal, but at much lower discrimination percentages during other sampling times. Metal oxide sensors require very high operating temperatures and voltages, thereby affecting the sensitivity of the device.

In order to overcome this, several combinations of metal oxide particles (especially WO_3_, which has high selectivity to acetone) with other nano-structures have been evaluated. Sensors consisting of SiO_2_ thin films doped with WO_3_ nanoparticles were able to selectively detect acetone concentrations as low as 20 ppb [101]. Xiong *et al.* [102] created CNT cantilevers with one end coated with WO_3_ and one end for single-molecule detection of acetone based on resonant frequency change. Photo-induced SWNT–TiO_2_ core/shell hybrid nanostructures showed room temperature fast and linear responses with the reversible detection of acetone vapors with concentrations in the few parts per million range [103]. Lu *et al.* [104] used an array of 32 sensors comprised of pristine, metal (Pd, Au)-decorated and polymer-decorated nanotubes to selectively detect acetone and NO_2_, HCN, HCl, Cl_2_ and benzene at ppm levels. MWNT-SnO_2_ sensors fabricated using the ultrasonic-assisted deposition-precipitation method was able to selectively detect sub-ppm levels of acetone, discriminating it from acetaldehyde and ethanol [105].

Unfortunately, the usefulness of acetone as a breath marker for diabetes is still controversial. An extensive review by Wang *et al.* [106] covering 3211 human subjects and 41 independent studies failed to establish a clear relationship between breath acetone and blood glucose level, the reason being that acetone is also a normal component of human metabolism and the detection of acetone is affected by several confounding factors, including:

Variation in acetone concentration in the general public
Measured on empty stomach or after meal
Nature and amount of food taken [107]
Breathing maneuvers alveolar *vs*. non-alveolar during sample collection [108]
Percentage of moisture in the breath
Before/after smoking
Presence of other disease conditions in patients (COPD, hypercapnia)
Exercise, sports, *etc*.
Significant acetone is produced only during extreme case of ketoacidosis.

An alternative solution was developed by Dillon *et al.* [109], where he monitored ^13^CO_2_ in the exhaled breath of individuals with normal glucose tolerance (NGT) and individuals with pre-diabetes and early-stage diabetes (PDED) during a ^13^C-labeled oral glucose tolerance test (OGTT) and found that glucose-derived breath ^13^CO_2_ was significantly lower in individuals with PDED compared with those with NGT from one to 3.5 h after glucose ingestion (*p* ≤ 0.05). A more promising approach was used by Grieter *et al.* [110], where they monitored eight VOCs using proton transfer reaction-mass spectrometry (PTR-MS) to differentiate 21 type 2 diabetic patients from 26 healthy controls with the sensitivity and specificity found to be 90% and 92%.

## 8. Conclusion and Future Work

The drastic rise in diabetic cases, as well as the cumbersome and invasive use of current diabetes monitoring tools has lead researchers to seek alternative diagnostic techniques. In this paper, we have detailed the different techniques that are currently being evaluated for non-invasive diagnostics. These techniques have been grouped into detection mediums as shown in Table 1 and their advantages and disadvantages grouped into Table 2. However, this is a rapidly evolving field, and new detection techniques are constantly being developed; for example, new optical techniques based on near-infrared spectroscopy (NIR) are being explored to detect glucose levels through the skin [111].

Non-invasive techniques to diagnose diabetes using saliva, sweat or tears have been explored as early as 1950, but only recently, due to the advent of nanomaterial sensors, has it been possible to develop portable devices that possess the required sensitivity. While several nanomaterials, ranging from metallic gold particles to polymer nano-composites, have been utilized, carbon nanotubes and, recently, graphene have shown the most promise as the materials of choice for future devices.

Finally, to achieve a wide use of these non-invasive technologies, the end sensing device needs to be developed at a very low cost to compete with the currently available blood glucose test strips. In the case of tear sensors, for example, a disposable sensing lens will need to have cost parity with current blood glucose strips or, alternatively, has to be reusable multiple times in order to make economic sense, especially in low and medium income countries. Ultimately, nanomaterial technology has to be addressed from cost-effective manufacturing methods and integration, which can challenge current low-cost test strip techniques.

**Table 1:** A list of the most studied nano-materials for detecting typical biomarkers and the relevant concentrations in various mediums. CNT, carbon nanotube; NP, nanoparticle.

Medium	Biomarkers	Typical Concentrations	Most Studied Materials
Blood	Glucose	2–30 mM [39]	ZnO [8,9,10] Metal NP [11,12,13,14] Metal oxide [112] CNT [16,17,19,20]
Urine	Glucose	2.78–5.5 mM [25]	Metal NP [113] Pt [26,114] CNT [28,29,115,116]
Saliva	Glucose	0.008–0.21 mM [38]	Polymer [40,41,46] Quantum dots [43,117] CNT [42,44,45] Graphene [118]
Sweat	Glucose	0.277–1.11 mM [49]	Polymer [59] CNT [56,119]
Tears	Glucose	0.1–0.6 mM [71]	Polymer [71,120] Graphene [76] Metal/metal oxide NP [121]
Breath	Acetone	21–0.5 ppm [93]	Polymer [95] Metal oxide [98,99,101] CNT [96,102,104]

**Table 2:** The advantages and disadvantages of using various media in diabetes diagnosis.

Media	Advantages	Disadvantages
**Blood**	Well-established analytical technique Low-cost instrumentation Continuous and reliable procedure	Invasive Highly uncomfortable for patients Infection risk from bruised skin
**Urine**	Non-invasive and painless Affordability Portable Rapid reproduction	Low accuracy Low glucose concentration levels Frequent calibration Susceptible to interference by bodily fluid volume to concentration ratio
**Saliva**	Non-invasive and painless Safe for children and adults Easy sample collection Cost-effective	Low concentration levels Requires high sensitivity and selectiveness to provide significant results Lag in saliva glucose, may not suitable for type I diabetes
**Sweat**	Easy sample collection Non-/minimally invasive Sufficient quantities and rapid reproduction	High calibration times Irritation and blistering of skin Inaccurate readings Lag and inconsistent testing Low glucose concentration levels
**Tears**	Highly accessible Less susceptibility to dilution Numerous testing methods Cost effective Continuously replenished	Poor correlation with blood glucose level Requires low detection limit and high sensitivity and selectivity Interference from high lactate levels and variable pH levels Lack of a suitable power source for testing
**Breath**	Non-invasive and painless Quick results Ease of use	Results and analysis influenced by multiple confounding factors Bio-markers are not very well defined
